# Hippocampal glutathione depletion with enhanced iron level in patients with mild cognitive impairment and Alzheimer’s disease compared with healthy elderly participants

**DOI:** 10.1093/braincomms/fcac215

**Published:** 2022-08-20

**Authors:** Pravat K Mandal, Anshika Goel, Ashley I Bush, Khushboo Punjabi, Shallu Joon, Ritwick Mishra, Manjari Tripathi, Arun Garg, Natasha K Kumar, Pooja Sharma, Deepika Shukla, Scott Jonathan Ayton, Amir Fazlollahi, Joseph C Maroon, Divya Dwivedi, Avantika Samkaria, Kanika Sandal, Kanu Megha, Sandhya Shandilya

**Affiliations:** National Brain Research Center, NeuroImaging and NeuroSpectroscopy Laboratory (NINS), Gurgaon, India; Florey Institute of Neuroscience and Mental Health, Melbourne, Australia; National Brain Research Center, NeuroImaging and NeuroSpectroscopy Laboratory (NINS), Gurgaon, India; Florey Institute of Neuroscience and Mental Health, Melbourne, Australia; Melbourne Dementia Research Centre, Melbourne, Australia; The University of Melbourne, Victoria, Australia; National Brain Research Center, NeuroImaging and NeuroSpectroscopy Laboratory (NINS), Gurgaon, India; National Brain Research Center, NeuroImaging and NeuroSpectroscopy Laboratory (NINS), Gurgaon, India; National Brain Research Center, NeuroImaging and NeuroSpectroscopy Laboratory (NINS), Gurgaon, India; All India Institute of Medical Sciences, New Delhi, India; Institute of Neurosciences, Medanta—The Medicity, Gurgaon, Haryana, India; Institute of Neurosciences, Medanta—The Medicity, Gurgaon, Haryana, India; Medanta Institute of Education and Research, Gurgaon, Haryana, India; National Brain Research Center, NeuroImaging and NeuroSpectroscopy Laboratory (NINS), Gurgaon, India; Florey Institute of Neuroscience and Mental Health, Melbourne, Australia; Melbourne Dementia Research Centre, Melbourne, Australia; The University of Melbourne, Victoria, Australia; Department of Radiology, University of Melbourne, Melbourne, Australia; Department of Neurosurgery, University of Pittsburgh Medical Center, Pittsburgh, USA; National Brain Research Center, NeuroImaging and NeuroSpectroscopy Laboratory (NINS), Gurgaon, India; National Brain Research Center, NeuroImaging and NeuroSpectroscopy Laboratory (NINS), Gurgaon, India; National Brain Research Center, NeuroImaging and NeuroSpectroscopy Laboratory (NINS), Gurgaon, India; National Brain Research Center, NeuroImaging and NeuroSpectroscopy Laboratory (NINS), Gurgaon, India; National Brain Research Center, NeuroImaging and NeuroSpectroscopy Laboratory (NINS), Gurgaon, India

**Keywords:** Alzheimer’s disease brain, glutathione, hippocampus, quantitative susceptibility mapping

## Abstract

Oxidative stress has been implicated in Alzheimer’s disease, and it is potentially driven by the depletion of primary antioxidant, glutathione, as well as elevation of the pro-oxidant, iron. Present study evaluates glutathione level by magnetic resonance spectroscopy, iron deposition by quantitative susceptibility mapping in left hippocampus, as well as the neuropsychological scores of healthy old participants (*N* = 25), mild cognitive impairment (*N* = 16) and Alzheimer’s disease patients (*N* = 31). Glutathione was found to be significantly depleted in mild cognitive impaired (*P* < 0.05) and Alzheimer’s disease patients (*P* < 0.001) as compared with healthy old participants. A significant higher level of iron was observed in left hippocampus region for Alzheimer’s disease patients as compared with healthy old (*P* < 0.05) and mild cognitive impairment (*P* < 0.05). Multivariate receiver-operating curve analysis for combined glutathione and iron in left hippocampus region provided diagnostic accuracy of 82.1%, with 81.8% sensitivity and 82.4% specificity for diagnosing Alzheimer’s disease patients from healthy old participants. We conclude that tandem glutathione and iron provides novel avenue to investigate further research in Alzheimer’s disease.

## Teaser

Mandal *et al*. measured left hippocampal glutathione using magnetic resonance spectroscopy together with iron deposition measured using quantitative susceptibility mapping of the same region. Significant differences in glutathione and iron levels were found in Alzheimer’s disease as compared with healthy old participants. Pro-oxidant iron has been examined with glutathione in the same group of population for the first time.

## Graphical Abstract

**Graphical abstract:**
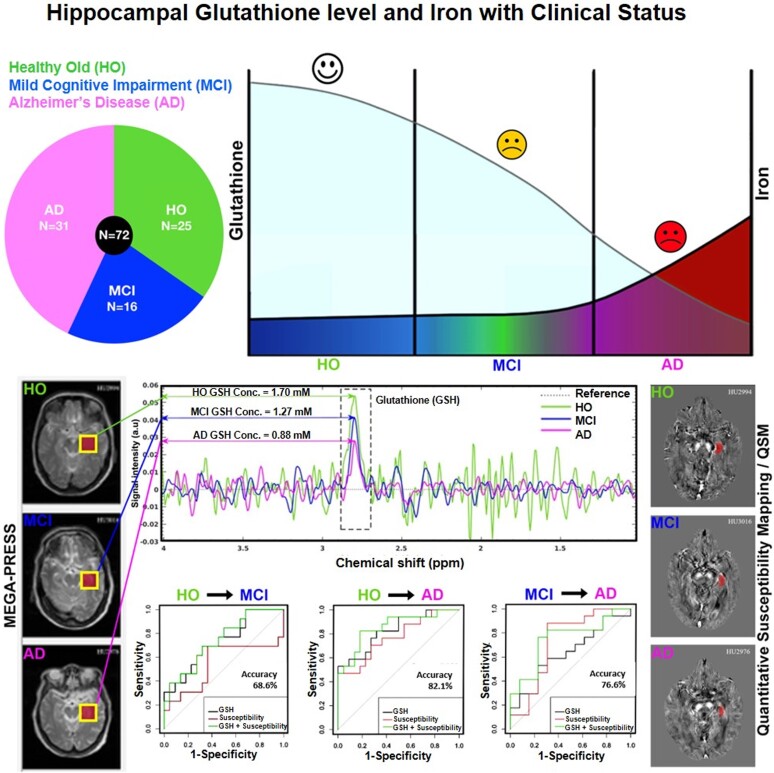

## Introduction

Alzheimer’s disease is characterized by gradual irreversible loss of memory and deterioration of cognitive function and daily activities.^1^ The histopathologic features of Alzheimer’s disease include synaptic degeneration, hippocampal neuronal loss, cortical deposition of extracellular amyloid plaques (Aβ) and intracellular neurofibrillary tangles. Despite its serious consequences and alarming incidence, pathogenesis of Alzheimer’s disease and disease-modifying therapeutic interventions are still under research.^2^

Oxidative stress (OS) and the role of antioxidant, glutathione (GSH) has been implicated in the pathophysiology of Alzheimer’s disease.^3,4^ GSH is a thiol-containing tripeptide (*γ*-glutamyl-cysteinyl-glycine) with an exposed highly reactive sulphydryl group (-SH), which neutralizes reactive oxygen and radicals. It is also involved in the chelation of metal ions.^5^ When oxidized, GSH takes the dimeric form of GSSG, which is inactive for radical scavenging and is restored to GSH via a redox cycle involving electron-acceptor NADPH and GSH reductase (EC 1.6.4.2).^6^

GSH is broadly distributed in the brain and can be quantified by magnetic resonance spectroscopy (MRS).^7,8^ MRS studies have revealed that GSH levels are significantly depleted in the hippocampal areas,^9^ frontal^9^ and cingulate cortices^10^ in mild cognitive impaired (MCI) and Alzheimer’s disease as compared with age-matched cognitively unaffected healthy old (HO) participants. GSH levels in the hippocampus and frontal cortices have also been found to be positively correlated with Mini-Mental State Examination (MMSE) scores.^9,11^ GSH depletion has also been confirmed by several post-mortem studies of the frontal lobe tissue in Alzheimer’s disease and MCI,^11^ the hippocampus in MCI,^12^ and the cingulate cortex in Alzheimer’s disease.^13^

Recent studies suggest higher cortical iron burden is associated with cognitive deterioration in the natural history of Alzheimer’s disease.^14,15^ The underlying mechanism is likely related to increased concentrations of the free cytoplasmic ferrous (Fe^2+^), which induces Fenton’s reaction to generate hydroxyl radicals (•OH) and lipid peroxidation, the signature of regulated cell death known as ferroptosis.^15^

GSH binds with cytoplasmic free iron, subsequently preventing ferrous iron from undergoing uncontrolled redox reactions which potentially causes oxidative damage.^16^ GSH suppresses iron toxicity by directly chelating metal ions and preventing them from undergoing redox cycling.^17^ It also acts as the substrate for the activity of GSH peroxidase 4, the checkpoint lipid peroxide-scavenging enzyme that blocks ferroptosis.^18^ Increased iron levels in tissue deplete GSH levels, possibly by direct oxidation.^16^

Quantitative susceptibility mapping (QSM) provides a specific marker for iron burden in the grey matter (GM).^19^ Increased iron deposition in hippocampal region using QSM predicted accelerated deterioration in composite (episodic memory) cognition test scores in amyloid-confirmed patients with Alzheimer’s disease.^14^ Whether iron accumulation in the hippocampal region actually increases in patients with MCI still remains to be determined as there are mixed evidences of increased QSM in MCI compared with HO.^20,21^ One study reported that iron deposition in hippocampal region measured using QSM has significant differences for HO, MCI and Alzheimer’s disease.^20^ They reported that iron deposition increases significantly for MCI and Alzheimer’s disease as compared with HO and increases significantly from MCI to Alzheimer’s disease. Another study reported no significant difference in iron deposition comparing either HO to MCI or MCI to Alzheimer’s disease, but a significant elevation was reported in Alzheimer’s disease compared to HO.^21^

To the best of our knowledge, no study has evaluated hippocampal GSH, iron, and neuropsychological scores simultaneously and analyzed the outcomes among the three study groups, HO, MCI and Alzheimer’s disease. We hypothesized that in Alzheimer’s disease, iron level increases and GSH decreases in the left hippocampus (LH) region. This might set up conditions conducive to oxidative damage and ferroptosis. To test this hypothesis, a cross-sectional study was conducted where we used MRS to assay hippocampal GSH together with iron deposition measured using QSM of the LH region and compared HO participants to MCI or Alzheimer’s disease patients.

## Methods

### Participant recruitment

A total of 72 participants (HO, *N* = 25; MCI, *N* = 16; Alzheimer’s disease, *N* = 31) were included in the study (Table 1). HO participants were recruited in collaboration with HelpAge India (National Capital Region, Delhi), whereas patients diagnosed with MCI and Alzheimer’s disease were recruited from the outpatient department, Department of Neurology by the two Neurologists, Dr. Manjari Tripathi (MD, DM), All India Institute of Medical Sciences, New Delhi and Dr. Arun Garg (MD, DM), Medanta, Gurgaon. MCI was diagnosed as per the revised Petersen criteria.^22^ Alzheimer’s disease was diagnosed as per the National Institute of Neurological and Communicative Disorders and Stroke (NINCDS) and the Alzheimer’s disease and Related Disorders Association (ADRDA) revised criteria.^23,24^ The eligibility criteria for all HO participants included ≥55 years of age. Participants with past or current psychiatric symptoms such as depression and/or anxiety; manic or psychotic episode, co-morbid alcohol or substance use disorder were excluded from the HO category. Participants with known contraindication for MRI (any metallic implants or claustrophobic) were also excluded from the study. The study was approved by the Institutional Human Ethics Committee at the National Brain Research Centre, Gurgaon. The purpose of the study was explained to all the participants and/or to the accompanying relatives before obtaining their written informed consent.

**Table 1:** Participant characteristics with outcome characteristic of GSH conc., susceptibility, SMMSE score, SMMSE memory recall score, CDR score, CDR memory score, CDT score, TMT-A (sec) and TMT-B (sec) among HO, MCI and Alzheimer’s disease group

Characteristics	HO (25)	MCI (16)	Alzheimer’s disease (31)	*P*-value*	Test statistics	Effect size
**Age (years)** ^a^	66.8 ± 5.30	68.06 ± 6.10	71.29 ± 7.70	**0**.**039**^b^	F(2) = 3.41	0.090
**Gender (M/F)**	12/13	12/4	18/13	0.230^c^	χ^2^ = 2.93	0.202
**GSH conc. (mM)** ^a^	2.17 ± 0.39 (25)	1.81 ± 0.35 (13)	1.58 ± 0.37 (26)	**<0**.**001**^b^	F(2) = 15.65	0.339
**Susceptibility (ppm)** ^a^	0.0064 ± 0.006 (22)	0.0061 ± 0.011 (16)	0.0140 ± 0.009 (22)	**0.009** ^b,f^	F(2) = 5.43	0.154
**SMMSE score** ^a^	29.40 ± 0.76 (25)	27.25 ± 6.15 (16)	16.66 ± 7.55 (29)	**<0**.**001**^d^	χ^2^ = 46.42	0.673
**SMMSE Memory recall**	2.56 ± 0.66 (25)	2.06 ± 0.68(16)	0.37 ± 0.68 (29)	**<0**.**001**^d^	χ^2^ = 47.15	0.683
**CDR score (0/0.5/≥1)**	22/0/0	1/13/1	1/4/23	**<0**.**001**^c^	χ^2^ = 91.47	0.839
**CDR Memory Score(0/0.5/≥1)**	22/0/0	1/13/1	1/3/24	**<0**.**001**^c^	χ^2^ = 95.59	0.858
**CDT score** ^a^	1.12 ± 0.33 (25)	2.00 ± 1.59 (16)	4.67 ± 1.58 (24)	**<0**.**001**^d^	χ^2^ = 38.99	0.609
**TMT-A (sec)** ^a^	47.76 ± 26.29 (25)	73.93 ± 51.14 (15)	133.47 ± 77.43 (15)	**0**.**001**^e^	F(2) = 6.17	0.233
**TMT-B (sec)** ^a^	106.52 ± 61.09 (25)	131.42 ± 75.38 (12)	318.50 ± 240.72 (8)	**0**.**047**^d^	χ^2^ = 6.12	0.139

a All continuous variables are represented as mean ± SD (*N*) and tested for normality using Shapiro–Wilk test and homoscedasticity using Levene’s test.

b One-way ANOVA test was used for testing significant difference between groups.

c χ^2^ test was used for testing significant difference between groups.

d Kruskal–Wallis rank test was used for testing significant difference between groups.

e Welch one-way ANOVA test was used for testing significant difference between groups.

f Variables are transformed using the Box-cox transform for normality assumption.

* All significant values were set at *P* < 0.05.

### Neuropsychological studies

Neuropsychological testing was performed prior to MRI and MRS scans. Clinical dementia rating (CDR) scale ^25^ was used to determine the severity of dementia symptoms. Cognitive functions were assessed using the Standardized Mini-Mental Status Examination (SMMSE)^26^ for assessing global cognition. Shulman’s clock drawing test (CDT)^27^ was performed to assess visuospatial functioning. Trail making test (TMT) Parts A and B^28^ were evaluated to assess visual attention, processing speed, and executive functioning. Higher CDR, CDT, and TMT signify enhanced cognitive impairment. To emphasize on memory measurements, SMMSE 5-item word list delayed recall score and CDR memory score were computed as an indirect measure of memory performance.

### MRI and MRS data acquisition

All ^1^H MRS on humans and phantom, and MRI data from human participants were acquired using a 3T MR scanner (Achieva, Philips, Netherlands), which was equipped with a dual-tuned (^1^H/^31^P) transmit/receive volume head coil (Rapid corporation, Germany) and 8 channel SENSitivity Encoding (SENSE) volume head coil.

### QSM data acquisition

Anatomical T_1_-weighted and gradient-echo scans were acquired using an 8-channel SENSE volume head coil. Three-dimensional Fast Field Echo (FFE) sequence was used with five echoes to generate phase and magnitude images to evaluate the susceptibility changes in the brain. Gradient-echo scans were performed using multi-FFE pulse sequence with following parameters: Field of view (FOV) = 240 × 200 × 160 mm^3^, voxel resolution = 1 × 1 × 1 mm^3^, echo time (TE) = 3.9 ms, echo time increment (δTE) = 5 ms, repetition time (TR) = 35 ms, no. of echoes = 5, no. of slices (with zero gap) = 160, resulting the scan time of 4 min 40 s. Three-dimensional T_1_-weighted image was acquired using Turbo Field Echo (TFE) sequence with TR = 8.4 ms, TE = 3.8 ms, no. of slices = 160, slice thickness = 1 mm, FOV = 228 × 180 × 160 mm^3^, flip angle = 8^°^, matrix = 228 × 92 × 160, and voxel resolution = 1 × 1 × 1 mm^3^, resulting in scan time of 5 min 11 s.

### GSH MEGA-PRESS data acquisition

All ^1^H MRI/MRS data from phantom as well as human participants were acquired using a dual-tuned (^1^H/^31^P) transmit/receive volume head coil. Both *in vivo* and freshly prepared phantom GSH data was acquired using MEGA-PRESS sequence. Scout images were collected in an axial plane for anatomical localization. Subsequently, for voxel placement, 2D T_2_-weighted MRI images with turbo spin echo sequence were acquired in three planes with the following acquisition parameters: TR = 3000 ms, TE = 80 ms, flip angle = 90°, turbo factor = 15, and zero slice gap. The voxel was placed at left hippocampal area (voxel size 2.5 × 2.5 × 2.5 cm3) using reference anatomical landmarks. The lower margin of the medial temporal region just above the skull base served as a reference to position MRS voxel in the LH region. For selective refocusing of the evolution of J-coupled GSH spins (H_β_ of cysteine) at 2.80 ppm, the editing pulse was set at 4.40 ppm for GSH spins (H_α_ of cysteine) and referred to as ‘ON’ as mentioned in the earlier published works.^7,9,29^ Off-resonance pulse was set at 5.00 ppm (referred to as ‘OFF’). Twenty interleaved spectral dynamics (each as an average of 16 number of spectral acquisitions) were acquired. Based on previous studies, the 90° excitation pulse and 180° refocusing pulse bandwidths were set at 2.4 kHz and 1.2 kHz, respectively, and other experimental parameters were set as follows: TE = 120 ms, TR = 2500 ms and SW = 2000 Hz.^7,9,29^ Water suppression was accomplished using Chemical Shift Selective Suppression pulse sequence,^30^ and excellent shimming of the voxel with water linewidth of ≤11–18 Hz was achieved using second-order pencil beam–volume in 3T Philips Scanner. MRS scan time for MEGA-PRESS scan was ∼14 min.

### QSM data processing

QSM data were processed using STI Suite version 3.0 in MATLAB 2018b and customized SUMEDHA package.^19,31^ Phase and magnitude images were extracted from the DICOM file, and a binary mask was created using Brain Extraction Tool (BET)^32^ using FSL.^33^ The raw-phase images of individual echoes were processed using STI Suite. The phases were unwrapped using a Laplacian-based algorithm.^34^ Background field was removed with the help of brain masks using Variable Sophisticated Harmonic Artifact Reduction for Phase data method.^35^ Iterative Least Square method was applied to process phase images of individual echoes separately to compute susceptibility maps.^36^ Average QSM image was further computed by averaging the susceptibility maps obtained for each echo.

**Figure 1:**
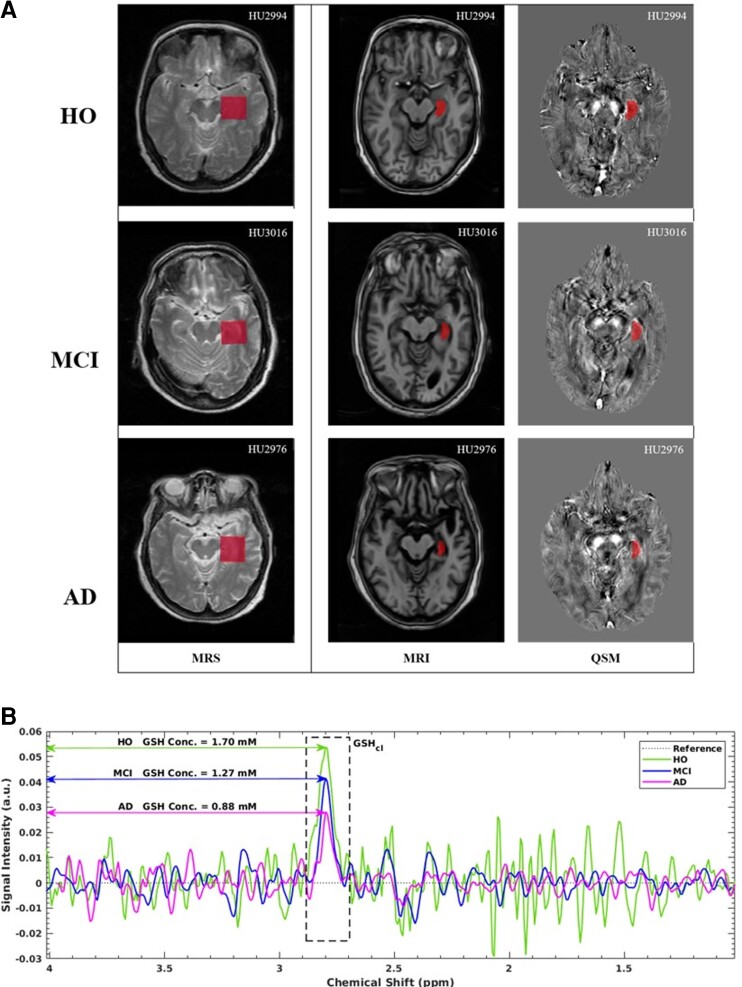
**Representative data of MRS, MRI and QSM.** (**A**) Illustrative axial slice for LH ROI during MRS, MRI and QSM for HO, MCI and Alzheimer’s disease participants. Voxel placement in LH region (red box) is shown during MRS. T_1_-weighted MRI image and QSM image shows the LH mask depicted in red colour. (**B**) Illustration of comparative amount of GSH peak obtained from LH region in the HO (green), MCI (blue) and Alzheimer’s disease (red) participants and the concentration values mentioned are PVC corrected

Susceptibility values for the specific region of interests (ROIs) were computed using the T_1_-weighted anatomical image. Initially, magnitude and T_1_-weighted images were bias-corrected using advanced normalization tools (ANTS) toolbox.^37^ Subsequently, bias-corrected files were denoised using ANTS. Denoised gradient magnitude image and T_1_-weighted image along with averaged QSM image were reoriented to a standard space using FSL.^33^ Subsequently, magnitude image was registered to T_1_-weighted image performed using the FLIRT (FMRIB’s Linear Image Registration Tool) using FSL. The registered T_1_-weighted image was segmented into anatomical regions using recon-all pipeline of Freesurfer.^38^ The LH region (severely affected in Alzheimer’s disease), cerebellum (relatively spared in Alzheimer’s disease), and middle frontal white matter (WM) regions (a reference region for QSM calculations) were separately extracted and converted to binary mask. All the masks were visually inspected for the correctness and were manually corrected using ITK-SNAP while being overlaid on QSM and T_1_-weighted images. To minimize the partial volume effects, the most inferior and superior slices of the masks and voxels of the tissue boundary were excluded. Individual binary masks were applied to QSM image, and the mean susceptibility of the region were computed. Figure 1A represents the segmented LH mask overlaid on T_1_-weighted MRI and QSM image for HO, MCI and Alzheimer’s disease participants. Susceptibility values from the ROIs were referenced to a middle frontal WM region as this region is known to be associated with the least susceptibility variation for HO, MCI and Alzheimer’s disease groups.^39^

### GSH MEGA-PRESS data processing

GSH MRS data obtained using MEGA-PRESS experiments were processed using in-house signal processing package KALPANA^10,40^ developed using MATLAB (The MathWorks); detailed data processing scheme has been elaborated in our earlier work.^9,40^ Briefly, the selective peak suppression of residual water (−70 Hz, 70 Hz) and lipid (−480 Hz, −520 Hz) peaks was accomplished using Hankel–Lanczos single value decomposition filtering method.^41^ Baseline noise was reduced using Gaussian and exponential filters. Singular spectrum analysis was employed for spectral baseline estimation, and subsequently, spectral fitting was accomplished using time and frequency domain non-linear least square cost function optimization.^9,42^  Figure 1B illustrates the comparative GSH peak in LH region among the HO, MCI and Alzheimer’s disease participants. GSH peak area from processed data was quantified to absolute concentration values (mM) using external reference (GSH phantom) as elaborated in our earlier work.^10^

### Absolute GSH quantitation

The absolute quantitation of *in vivo* GSH was performed with external calibrated referencing using GSH phantom-based in our earlier work.^10^

(1) $$\begin{array}{rl} {}[Absolute\;Conc_{GSH}]= & \;\frac{Area_{GSH}-k}{m}\times \frac{1-\exp\;\left(-\;\frac{TR}{T1_{Phantom}}\right)}{1-\exp\;\left(-\;\frac{TR}{T1_{GSH}}\right)}\\ & \times \frac{\exp\;\left(-\;\frac{TE}{T2_{Phantom}}\right)}{\exp\;\left(-\;\frac{TE}{T2_{GSH}}\right)} \end{array}.$$

The relaxation times (T_1_ and T_2_) of GSH, both for phantom and brain tissue were inferred from published literature using 3T Philips scanner, details of which have been elaborated in our earlier work.^10^ Existing literature reports negligible GSH levels within CSF, with the GSH signal principally emerging from GM and WM brain tissues.^43^ To eliminate the effect of increased CSF or tissue degeneration within the MRS voxel, a partial volume correction (PVC) methodology was employed based on our earlier work.^10^ The brain tissue segmentation of the MRI was performed using SPM12 (Wellcome Centre for Human Neuroimaging at University College London) and calculation of PVC correction factors were performed using the KALPANA package.^40^

### Statistical analysis

The characteristics (age) of participants and outcome measures (GSH concentration, susceptibility value, and neuropsychological scores) on a continuous scale have been summarized using mean ± SD, whereas categorical variable (gender) have been reported as male (M) to female (F) ratio. Age, GSH concentration, susceptibility values and neuropsychological scores were assessed for normality using sample size, mean, SD, skewness and homoscedasticity using Levene’s test. Box-Cox transformation has been applied for non-normally distributed outcome variables; (susceptibility, SMMSE, CDT and TMT-B). Among these variables, only susceptibility values satisfy the normality assumption. The χ^2^ test was used to assess categorical variables, gender and CDR scores. One-way analysis of variance (ANOVA) test was performed for evaluating difference in age, GSH concentration and susceptibility among the groups of participants (HO, MCI, and Alzheimer’s disease). Differences in SMMSE score, CDT score and TMT-B among participant groups were assessed by a non-parametric test, Kruskal–Wallis test. TMT-A scores were assessed by Welch one-way ANOVA test due to the non-homogeneous nature of data. Pairwise comparison was computed using the Tukey–Kramer *post hoc* test.

Moreover, for assessing the diagnostic utility of GSH concentration and susceptibility value over the LH region to differentiate the three study groups (HO, MCI and Alzheimer’s disease), receiver-operating characteristic (ROC) analyses were performed. Multivariate ROC curve analysis was also performed using the predicted probability values obtained by binary logistic regression for the combined effects of GSH concentration and susceptibility value changes. For each ROC curve, the area under the curve (AUC), sensitivity, specificity and accuracy were reported. Two-tailed significance levels for all the statistical analyses were set at *P* < 0.05. Most statistical analyses were performed using MedCalc software (version 15.8) and verified with the NINS-STAT, a MATLAB-based in-house statistical toolbox. Linear regressions were performed using Prism 9.0 (Graphpad). The outcome variables were CDT and CDR scores, whereas the predictor variable was either iron or GSH levels. The models were collapsed across diagnoses since the sample size was too small to permit analysis of separate diagnoses.

### Data availability

The data that support the findings of this study are available on request from the corresponding author.

## Results

The demographic information along with metabolite concentration, susceptibility, and neuropsychological measurements of the participant groups are presented in Table 1. The mean age of total 42 males and 30 females (*N* = 72) was 69.01 ± 6.83 years.

QSM data for three HO participants were not included in the analysis owing to their poor image quality. QSM data for nine Alzheimer’s disease patients were excluded as two did not complete the acquisition session and seven data were of poor quality. A total of 60 participants (HO, *N* = 22; MCI, *N* = 16; Alzheimer’s disease, *N* = 22) were included in the QSM study. MRS data (GSH) of three MCI patients were not included in the analysis as they did not complete the acquisition session. Five MRS data in the Alzheimer’s disease group were excluded as four were not present for the acquisition session and one image was of poor data quality. A total of 64 participants (HO, *N* = 25; MCI, *N* = 13; Alzheimer’s disease, *N* = 26) were included in the MRS study.

**Figure 2:**
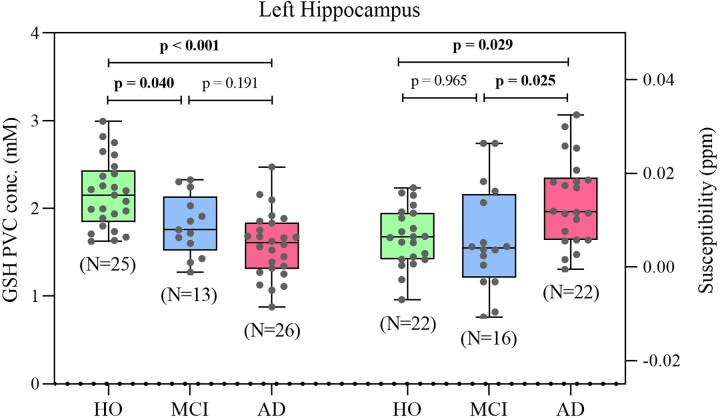
**GSH and iron level in HO, MCI and Alzheimer’s disease**. Box plot of LH GSH concentration measured using MRS (left Y axis) and associated susceptibility with iron as dominant source measured using QSM (right Y axis) from each clinical cohort. Statistical analysis was done using one-way ANOVA test followed by Tukey–Kramer for *post hoc* multiple comparison

To assess the GSH level in HO, MCI and Alzheimer’s disease, we analyzed the mean ± SD of GSH PVC concentration in LH region for each of the three cohorts (Table 1). The mean GSH concentration was significantly different among HO, MCI, and Alzheimer’s disease groups (*P* < 0.001). Pairwise comparisons between the groups revealed a significant mean difference in GSH in the LH for both Alzheimer’s disease (*P* < 0.001) and MCI (*P* = 0.018) as compared with HO, whereas no difference (*P* = 0.191) was observed between MCI and Alzheimer’s disease (Fig. 2). In tandem, significant change was detected in mean susceptibility (suggestive of iron level) in the LH region among HO, MCI and Alzheimer’s disease groups (*P* < 0.05). *Post hoc* analysis revealed iron levels in Alzheimer’s disease was different than HO (*P* = 0.029) and MCI (*P* = 0.025) (Fig. 2). However, iron in the LH region for MCI was not significantly different to HO (*P* = 0.965). No significant differences were reported for iron levels in the cerebellar region (Supplementary Figure 1).

GSH and susceptibility level differences were also assessed using age as a covariate, as age differs across the groups (Table 1). Pairwise comparisons between the groups showed significant lower levels of GSH in both MCI (MD = 0.36, *P* = 0.006) and Alzheimer’s disease (MD = 0.592, *P* < 0.001) as compared with HO, where MD is a mean difference value (Table 2). Similarly, pairwise comparisons between the regions revealed significant higher susceptibility levels in Alzheimer’s disease as compared with HO (MD = 0.006, *P* = 0.023) and MCI (MD = 0.007, *P* = 0.013) (Table 2).

**Table 2:** *Post hoc* analysis of (a) GSH and (b) susceptibility after adjustment of age

Group comparison	Mean difference	S.E. of difference	t-statistic	*P*-value
**(a) GSH conc.**				
**Alzheimer’s disease versus HO**	–0.592	0.108	–5.472	<0.001
**Alzheimer’s disease versus MCI**	–0.232	0.129	–1.789	0.074
**HO versus MCI**	0.36	0.130	2.765	0.006
**(b) Susceptibility**				
**Alzheimer’s disease versus HO**	0.006	0.003	2.277	0.023
**Alzheimer’s disease versus MCI**	0.007	0.003	2.460	0.013
**HO versus MCI**	0.001	0.003	0.353	0.724

Based on estimated marginal means.

**Figure 3:**
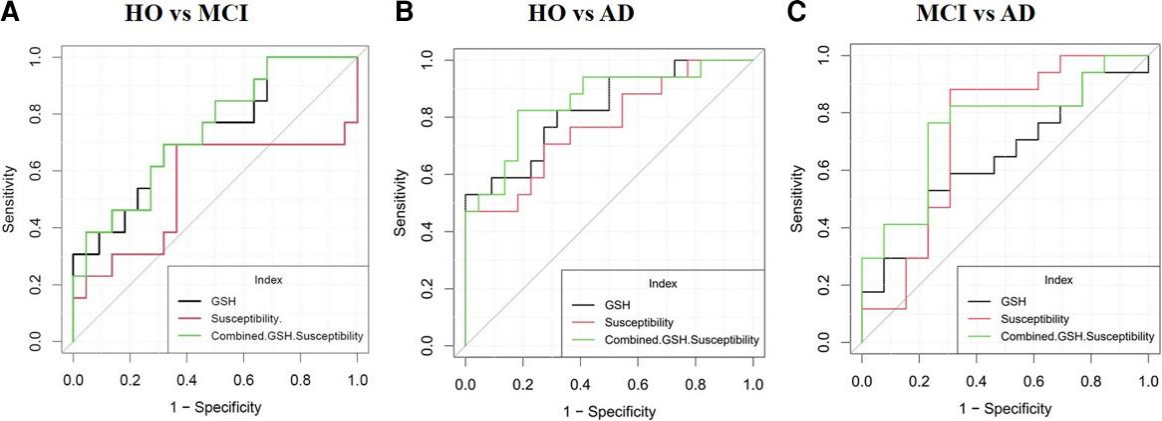
**Receiver operator characteristic analysis multivariate ROC curves demonstrating the predictive utility of GSH (black), iron (red) and their combined effects (green) for disease diagnosis of MCI and Alzheimer’s disease.** (**A**) GSH can predict MCI with respect to HO with 0.738 AUC, 0.720 sensitivity and specificity of 0.692. Iron predicts MCI with 0.551 AUC, 0.636 sensitivity and specificity of 0.688. Combined iron and GSH have 0.741 AUC, 0.692 sensitivity and 0.682 specificity. (**B)** GSH predicts Alzheimer’s disease with respect to HO with 0.868 AUC, 0.760 sensitivity and specificity of 0.846. Iron predicts Alzheimer’s disease with 0.744 AUC, 0.682 sensitivity and specificity of 0.682. Combined iron and GSH have 0.856 AUC, 0.818 sensitivity and 0.824 specificity. (**C)** GSH can predict Alzheimer’s disease with respect to MCI with 0.675 AUC, 0.615 sensitivity and specificity of 0.692. Iron predicts Alzheimer’s disease with respect to MCI with 0.722 AUC, 0.864 sensitivity and specificity of 0.688. Combined iron and GSH have 0.751 AUC, 0.769 sensitivity and 0.765 specificity

ROC analysis was performed to assess the diagnostic utility of GSH concentration and iron level in LH region and their combined effect to distinguish MCI and Alzheimer’s disease from HO and from each other (Fig. 3, Table 3). Iron levels were differentiating Alzheimer’s disease from HO group (*P* = 0.006) with 0.744 AUC, 68.2% accuracy and MCI from Alzheimer’s disease (*P* = 0.021) with 0.722 AUC and 80.4% accuracy. Meanwhile, GSH levels showed slight improved classification of MCI from HO group (*P* = 0.017) with 0.738 AUC, 70.9% accuracy and Alzheimer’s disease from HO group (*P* = 0.0001) with 0.868 AUC, 80.8% accuracy. However, GSH levels were not potentially differentiated in MCI and Alzheimer’s disease group (*P* = 0.079) and iron levels were not discriminating HO and MCI groups (*P* = 0.595). Higher accuracy was observed by combining GSH and iron levels in Alzheimer’s disease from HO participants with 82.1% accuracy (*P* = 0.000). Fig. 3 depicts the diagnostic accuracy tests conducted using ROC curves for independent GSH, iron, and their combined effect to differentiate HO, MCI and Alzheimer’s disease. Furthermore, memory measures from neuropsychology tests associated more with the GSH. The association of GSH with memory recall (*r* = 0.44) improved in comparison to the SMMSE total score (*r* = 0.307).

**Table 3:** Diagnostic accuracy characteristics (AUC, sensitivity, specificity and accuracy) for GSH concentrations, susceptibility, and the combined effect of GSH concentration and susceptibility levels to differentiate among the three groups (HO, MCI and Alzheimer’s disease)

Parameter	AUC	S.E.	*P*-value	Sensitivity	Specificity	PPV	NPV	Accuracy
**HO versus MCI**								
**GSH conc.**	0.738	0.086	0.017*	0.720	0.692	0.785	0.612	0.709
**Susceptibility**	0.551	0.104	0.595	0.636	0.688	0.761	0.547	0.656
**GSH + susceptibility**	0.741	0.085	0.018*	0.692	0.682	0.582	0.776	0.686
**HO versus Alzheimer’s disease**								
**GSH conc.**	0.868	0.049	0.000*	0.760	0.846	0.799	0.814	0.808
**Susceptibility**	0.744	0.075	0.006*	0.682	0.682	0.727	0.633	0.682
**GSH + Susceptibility**	0.856	0.062	0.000*	0.818	0.824	0.789	0.849	0.821
**MCI versus Alzheimer’s disease**								
**GSH Conc.**	0.675	0.091	0.079	0.615	0.692	0.507	0.777	0.666
**Susceptibility**	0.722	0.091	0.021*	0.864	0.688	0.843	0.723	0.804
**GSH + susceptibility**	0.751	0.090	0.020*	0.769	0.765	0.628	0.865	0.766

*All significant values are set to *P* < 0.05.

## Discussion

Previously conducted independent studies have reported the association of Alzheimer’s disease pathology with GSH depletion^9,11^ and iron burden.^14,20,21^ To the best of our knowledge, this is the first study to investigate the GSH concentration and iron level in the LH region, a characteristically affected area in Alzheimer’s disease.

LH was the ROI studied by tandem MRS and QSM imaging because it is more impacted in Alzheimer’s disease than the right hippocampus.^44–46^ The hippocampus is primarily involved in memory formation.^47^ In patients with Alzheimer’s disease, hippocampal atrophy and LH volume loss have been correlated with poor performance on memory tasks,^48^ cognitive impairment^49^ and reduced neuropsychological performance compared with healthy controls.^50^ One study reported significant reduction in LH volume (–11%, *P* = 0.02) in patients with MCI as compared with controls but no differences were reported in right hippocampal volume (–4%).^44^ In addition to the atrophy, LH differs significantly in shape between patients with Alzheimer’s disease and HO^45^ and is reportedly more informative in diagnosis of amnestic MCI than the right hippocampal region.^46^

GSH is present in the mM range in brain tissue.^51^ Our findings provide *in vivo* confirmatory evidence to the previously conducted post-mortem human studies^11,13,52^ that have reported Alzheimer’s disease -associated brain GSH depletion. We previously reported that GSH levels are unaltered in the cerebellum but are significantly depleted in both hippocampus and frontal cortex in Alzheimer’s disease compared with HO.^9^ The present study corroborates these findings and aligns them with changes in iron levels in the same regions (Supplementary Figure 1).

Iron is the most abundant transition metal in human body and plays several essential functions including oxygen transport, mitochondrial respiration, protein and DNA synthesis, myelination, dendrite development and neurotransmitter biosynthesis.^53^ QSM is an *in vivo* MRI technique that has been validated for its ability to selectively quantify iron burden in GM of brain.^19,34^ Several groups have used QSM for investigating the impact of brain iron burden on the natural history of Alzheimer’s disease.^20,21^ Notably, higher iron levels in hippocampal region predict accelerated deterioration in composite cognition tests for episodic memory.^14^ In agreement with our findings, one research group observed significantly increased hippocampal susceptibility in Alzheimer’s disease compared with HO.^20^ Based on the ROC analysis, iron deposition measured using QSM were concluded to be significantly different between HO and MCI (*P* = 0.018) or Alzheimer’s disease (*P* < 0.0001).^20^ In another study, iron levels were reported to be significantly increased in the hippocampal region in Alzheimer’s disease compared with HO.^21^

Iron distribution in the adult human brain is quite heterogeneous as assessed by post-mortem measurements.^54^ Our QSM findings are in agreement with an autopsy study that reported significantly higher iron levels in the hippocampus (288 ± 20 mg/g dry weight in Alzheimer’s disease, *N* = 10; 216 ± 16 mg/g dry weight in HO, *N* = 11), but no change was observed in the cerebellum for Alzheimer’s disease in comparison to HO (306 ± 54 mg/g dry weight in Alzheimer’s disease, *N* = 10; 297 ± 28 mg/g dry weight in HO, *N* = 11).^55^ A meta-analysis of seven studies also indicated a trend (*P* = 0.056) of increased iron levels in the hippocampal areas of autopsy brains in Alzheimer’s disease.^56^ No change was observed in the iron levels in the cerebellum.

GSH binds cytoplasmic free iron, subsequently preventing ferrous iron from undergoing uncontrolled redox reactions potentially causing oxidative damage.^16^ A chemical shift of cysteine of GSH β-H from 2.80/2.95 ppm to 3.22 ppm has been attributed to GSH-iron complexation and subsequent oxidation of GSH to GSSG.^16,57^

A recent large study on post-mortem tissue proposed that the level of tissue iron is a trait that influences the probability of neurodegeneration in Alzheimer’s disease by ferroptosis, a regulated cell-death pathway that is initiated by signals such as GSH depletion and lipid peroxidation.^15^ This underscores the synergistic interplay between increased iron burden and decreased GSH levels, which would bring about more lipid peroxidation and cell death.

In the present study, MRS technique is used to assay left hippocampal GSH together with iron deposition measured using QSM of the same region. Significant differences in GSH and iron levels were found in Alzheimer’s disease as compared to HO. GSH depletion is said to be an early event in progression of Alzheimer’s disease,^22,58^ but to the best of our knowledge, pro-oxidant iron has been examined with GSH in the same group of population for the first time.

## Limitations

This study is based on cross-sectional data and therefore does not address direction or sequence of events for the association of GSH and iron with Alzheimer’s disease pathology and their causal process. The results presented in this study are also limited by a modest sample size and cross-sectional study design, therefore there is a need for replication in a study with a larger cohort to qualitatively assess the causality of Alzheimer’s disease progression with GSH and iron alterations. Finally, it is to be noted that our hippocampal voxels contained the substantial fraction of extraneous non-hippocampal tissue due to technological limitation. Thus, presented GSH values are exclusively from the hippocampal region plus small amount from outside hippocampal region due to voxel size.

## Supplementary Material

fcac215_Supplementary_Data

## Abbreviations

AUC =: area under the curve
CDR =: Clinical Dementia Rating
CDT =: clock drawing test
FFE =: Fast Field Echo
GSH =: Glutathione
HO =: healthy old
LH =: left hippocampus
MCI =: mild cognitive impairment
MRS =: magnetic resonance spectroscopy
QSM =: quantitative susceptibility mapping
OS =: oxidative stress
SMMSE =: Standardized Mini-Mental Status Examination
TE =: echo time
TR =: repetition time
TMT =: trail making test
