# Supplementary material for: Hippocampal glutathione depletion with enhanced iron level in patients with mild cognitive impairment and Alzheimer’s disease compared with healthy elderly participants
Source: Brain Commun. 2022 Aug 20;4(5):fcac215. doi: 10.1093/braincomms/fcac215 (PMC9445173; doi:10.1093/braincomms/fcac215)
Supplement: fcac215_Supplementary_Data [file fcac215_supplementary_data.docx]

# Supplementary Material


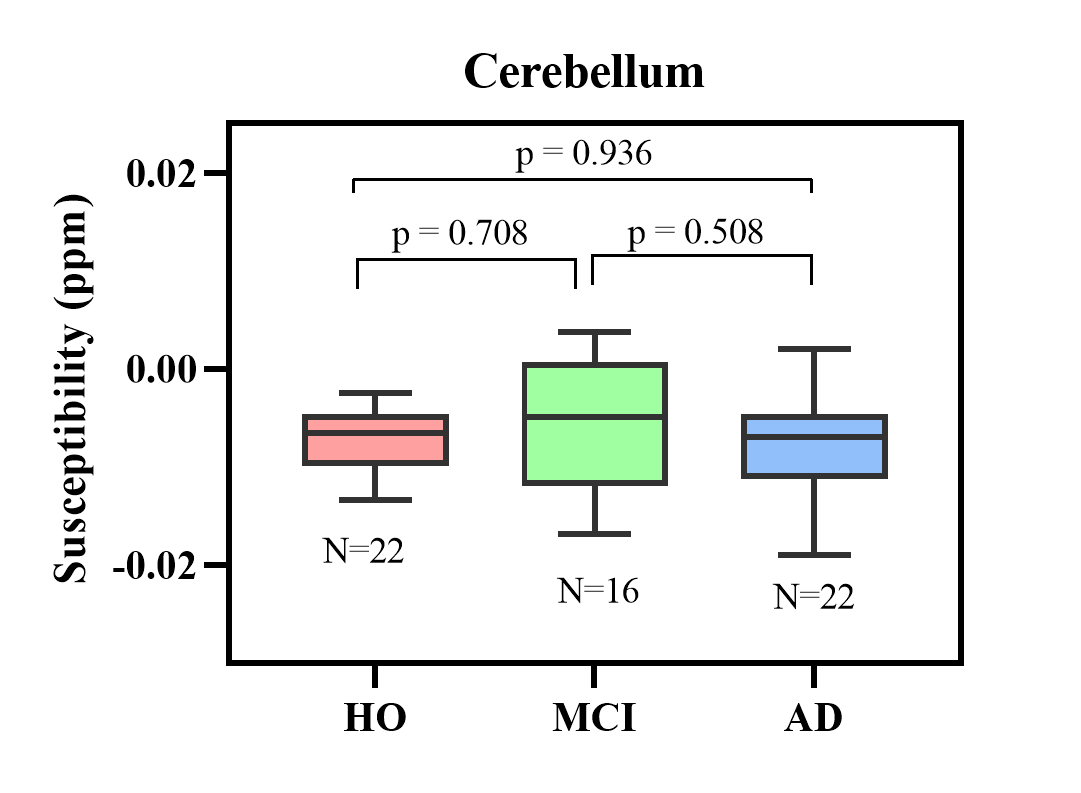


**Supplementary Figure 1:** Box plot for susceptibility values in the cerebellar region. There are no significant changes in susceptibility values for HO, MCI, and AD participants.
